# Amplified fragment length homoplasy: *in silico *analysis for model and non-model species

**DOI:** 10.1186/1471-2164-11-287

**Published:** 2010-05-07

**Authors:** Margot Paris, Benjamin Bonnes, Gentile Francesco Ficetola, Bénédicte N Poncet, Laurence Després

**Affiliations:** 1 Laboratoire d'Ecologie Alpine, CNRS-UMR 5553, Université Joseph Fourier, BP 53, 38041 Grenoble Cedex 09, France

## Abstract

**Background:**

AFLP markers are widely used in evolutionary genetics and ecology. However the frequent occurrence of non-homologous co-migrating fragments (homoplasy) both at the intra- and inter-individual levels in AFLP data sets is known to skew key parameters in population genetics. Geneticists can take advantage of the growing number of full genome sequences available for model species to study AFLP homoplasy and to predict it in non-model species.

**Results:**

In this study we performed *in silico *AFLPs on the complete genome of three model species to predict intra-individual homoplasy in a prokaryote (*Bacillus thuringiensis *ser. *konkukian*), a plant (*Arabidopsis thaliana*) and an animal (*Aedes aegypti*). In addition, we compared *in silico *AFLPs to empirical data obtained from three related non-model species (*Bacillus thuringiensis *ser. *israelensis, Arabis alpina *and *Aedes rusticus*). Our results show that homoplasy rate sharply increases with the number of peaks per profile. However, for a given number of peaks per profile, genome size or taxonomical range had no effect on homoplasy. Furthermore, the number of co-migrating fragments in a single peak was dependent on the genome richness in repetitive sequences: we found up to 582 co-migrating fragments in *Ae. aegypti*. Finally, we show that *in silico *AFLPs can help to accurately predict AFLP profiles in related non-model species.

**Conclusions:**

These predictions can be used to tackle current issues in the planning of AFLP studies by limiting homoplasy rate and population genetic estimation bias. ISIF (In SIlico Fingerprinting) program is freely available at http://www-leca.ujf-grenoble.fr/logiciels.htm.

## Background

Many key questions in evolutionary genetics and ecology cannot be addressed solely using model species, and until recently, the genomic study of non-model species (ecogenomics [1,2]) was limited by the lack of genomic information available. However, the taxonomical range of model species for which whole genome sequences are readily available in databases is already wide and is rapidly expanding. The genomic resources already available can constitute a key tool for molecular ecologists, to optimize experimental design and decrypt the genetics of related non-model species [3,4]. Amplified Fragment Length Polymorphism (AFLP) [5] is one of the most extensively used DNA fingerprinting methods and has many applications on model and non-model species, such as inferring genetic structure, genetic diversity, demography, phylogeny, genotyping, gene mapping or genome scan analyses [6-11]. Genomic DNA is digested into thousands of fragments using restriction enzymes. A subset of the genomic restriction fragments is PCR amplified using primers with 1 - 4 selective bases each, thereby reducing the number of fragments on the profile. The fragments are separated lengthwise using electrophoresis, and discrete peaks can be visualized on a typical AFLP profile. Each discrete peak position is scored, i.e. characterized as a biallelic locus (coded 0/1) in a 50 - 500 bp range [11]. This technique is based on the assumption that co-migrating fragments of the same length are homologous and come from the same chromosomal region. In practice, this assumption is not systematically tested [12].

The quality of the AFLP result (i.e. the profile) is determined by several factors, including the number of peaks detected and their length distribution. The AFLP method usually produces 40 - 200 peaks per profile [7]. A large number of peaks in a profile increases the probability of detecting genetic polymorphism, but also the probability of poorly separated unscorable peaks and homoplasy. Homoplasy occurs when non-identical fragments originating from different loci in the genome co-migrate [12].

Peak homoplasy can arise through two major processes in AFLP data sets. First, at the individual level, an AFLP peak can contain several non-identical co-migrating fragments which co-migrate by chance, or because they share similar sequences but not the same location in the genome (repeated DNA). These fragments with high similarity could be orthologous or paralogous copies of genes, pseudogenes, transposable elements, or repetitive sequences with unknown functions [13]. Second, at the inter-individual level, AFLP peaks of the same length in two different profiles are not necessarily homologous [13-15]. Additionally, homoplasy between individuals can be increased artificially during the scoring. This "technical homoplasy" has recently been described by Arrigo et al. [16] which recommended AFLP scoring procedures minimizing this bias. Caballero et al. [17] recently used a theoretical approach to estimate biases due to co-migrating fragments in population genetic analyses based on AFLP data. They found that inter-individual homoplasy causes overestimation of allele frequencies, underestimation of the degree of differentiation between subpopulations and reduces the performance of genomic scan when detecting loci under selection. Furthermore, intra-individual homoplasy can also affect the estimation of genetic estimators [18,19]. Evaluating and limiting homoplasy in data sets used for population genetic inference and genome scan analysis is therefore of primary importance.

Few studies have attempted to estimate the proportion of co-migrating AFLP fragments in a profile or between individuals either directly by sequencing peaks [15,20-23], or indirectly by comparing AFLP patterns resulting from several runs of selective amplification using primers with an additional nucleotide [14,24], by modeling the fragments' length distribution [12,25] or by performing *in silico *AFLP [13,26]. These studies established that homoplasy is frequent in most AFLP data sets and is higher in short peaks or when many AFLP peaks are generated. In the rare studies evaluating the proportion of co-migrating fragments, homoplasy ranged from 4% in individuals up to 100% across species from distantly related taxa.

At the individual level, the number of peaks and the clarity of the profiles are strongly dependent on the selection of restriction enzymes and on the number and the sequence of selective bases. For most studies on plant and animal genomes, the restriction enzyme combination EcoRI/MseI and three selective bases for each primer are used [7,27]. For larger or polyploid genomes, a two-step amplification procedure using 4 selective bases is recommended [28,29]. On the other hand, for small bacterial and fungal genomes, a single amplification with one or two selective nucleotides is sufficient [30,31]. Moreover, the nucleotide composition of the selective bases influences the number of amplified fragments and their length distribution [26,32] thereby affecting homoplasy. For example, the use of A/T selective bases results in an over-representation of shorter fragments in *Arabidopsis thaliana *[26].

Although all these factors have an important influence on the AFLP profiles, it is difficult to foresee which combinations of enzymes and selective bases will be the most informative for the AFLP study of non-model species. The most commonly used method is to first test a large amount of primer combinations on a sub-sample of representative individuals before performing the whole population analysis using a few selected primer combinations, but this process can be time consuming and expensive. Another approach is the use of bioinformatics, to take advantage of the exponentially growing number of full genome sequences available, for example by performing *in silico *AFLPs [33-35].

*In silico *AFLPs simulate the AFLP experimental process on a full sequenced genome and provides the length of the virtual fragments, their sequences and their positions along the genome at no cost. In this study, we first validated this approach by showing the consistency between *in silico *and experimental AFLPs on the model species *Arabidopsis thaliana*. We also examined whether the fluorescence intensity of peaks was a reliable predictor of homoplasy. Then, *in silico *AFLPs were performed on three model species genomes covering wide taxonomical and genome size ranges, including a prokaryote (*Bacillus thuringiensis *ser. *konkukian*, 5.2 Mb), a plant (*Arabidopsis thaliana*, 120 Mb) and an animal genome (*Aedes aegypti*, 1,310 Mb). This made it possible to compare the profile quality (number of peaks generated and proportion of non-homologous co-migrating fragments per profile) in genomes which widely differ in size and in the abundance of repetitive sequences [36-38], but comparable for their GC content (35.4%, 36% and 38.2% respectively). We first examined the effect of genome features such as size and repeated elements prevalence and AFLP parameters (number of selective bases, GC content) on homoplasy at the intra-individual level (i.e. co-migrating fragments within a peak). We then looked at the effect of these parameters on the number of peaks generated and peak length distribution. As most AFLP studies are used on non-model species, we finally asked to what extent knowledge of the profiles obtained *in silico *for model species can help to predict the quality of the profiles obtained empirically on non-model related species. To answer this question, we compared the *in silico *AFLP results obtained in the three model species with the experimental AFLP profiles obtained in three related, non-model species (*Bacillus thuringiensis *ser. *israelensis*, *Arabis alpina *and *Aedes rusticus*).

## Results

### The ISIF procedure: description and validation on model species

The user friendly program ISIF allows carrying out *in silico *AFLPs on species for which whole genome sequences are available. ISIF is freely available at http://www-leca.ujf-grenoble.fr/logiciels.htm. The program can analyze all sequences saved as plain text, without line numbers and spaces, such as text files. The AFLP procedure is simulated by the program step by step: 1) identification of the restriction sites and production of the pool of restriction fragments, 2) selection of the final set of fragments that exhibit the selective bases used for the amplification, and 3) determination of the length of all peaks in the AFLP profile by adding the adaptor length to the selective AFLP fragments. For any restriction enzyme and selective bases combinations, ISIF can provide the sequences of the virtual fragments, their positions along the genome, their length and the length of the associated peaks in the AFLP profile.

**Figure 1:**
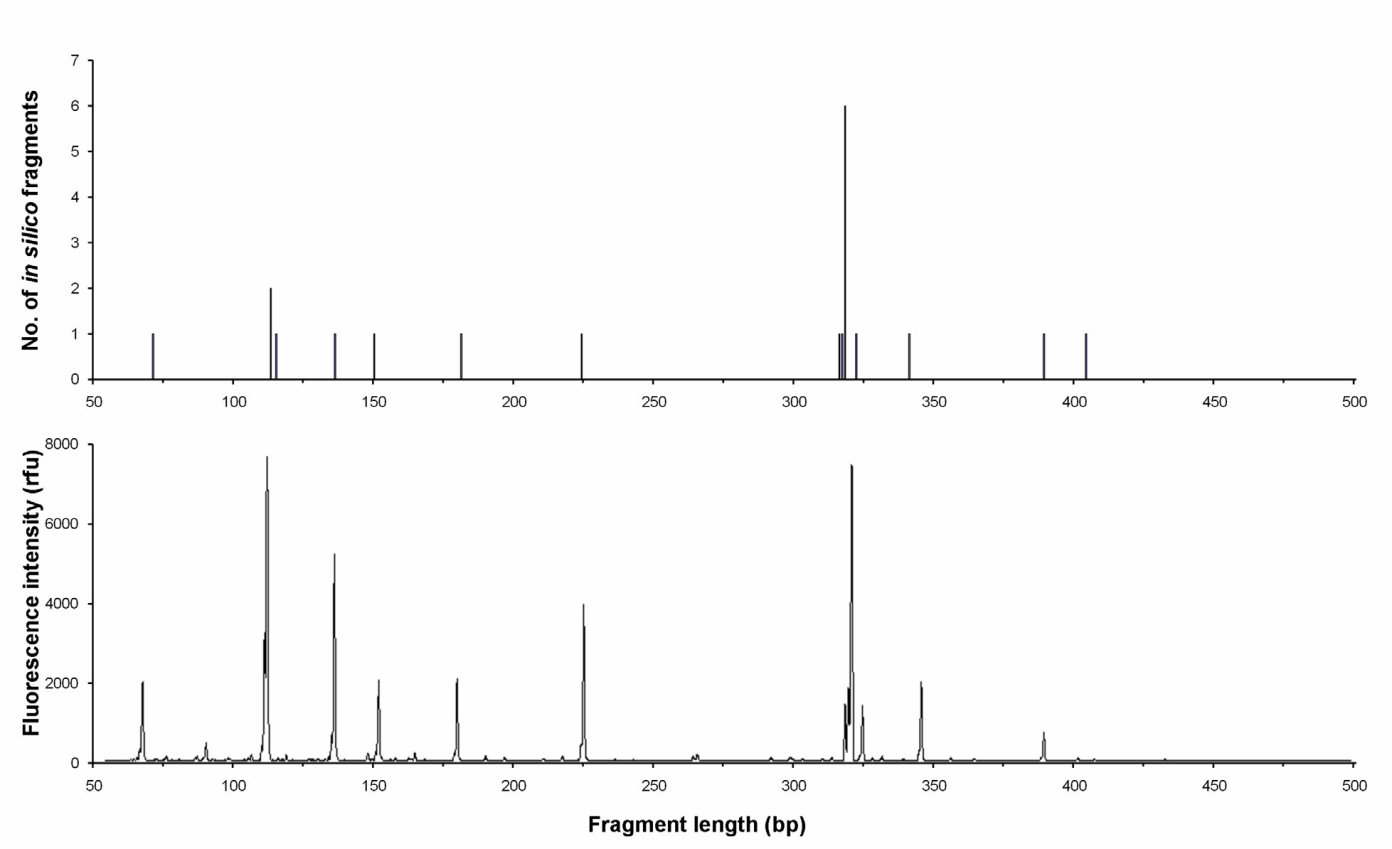
**Comparison of the *in silico *(upper panel) and experimental (lower panel) AFLP profiles obtained with the primer combination EcoRI+ATG/MseI+ATG for the model species *Arabidopsis thaliana***.

*In silico *AFLPs on *A. thaliana *using the primer combination EcoRI+ATG/MseI+ATG generated 20 non-identical fragments between 50 and 500 pb; however, due to size homoplasy, this only corresponded to 13 different peak sizes (Figure 1). Experimental AFLP generated a profile with 12 peaks (Figure 1) and the two profiles almost perfectly matched, except for the expected peak at 410 bp that was scored as missing in the experimental profile (Figure 1) because it was below the detection threshold (only 85 rfu in intensity). We repeated the whole AFLP protocol three times, and we found no difference between the three experimental AFLP profiles; the reproducibility rate was 100%. All experimental sequenced fragments obtained by pyrosequencing perfectly matched the sequences of *in silico *fragments, including the 410 bp fragment.

### *In silico *AFLP profiles

**Figure 2:**
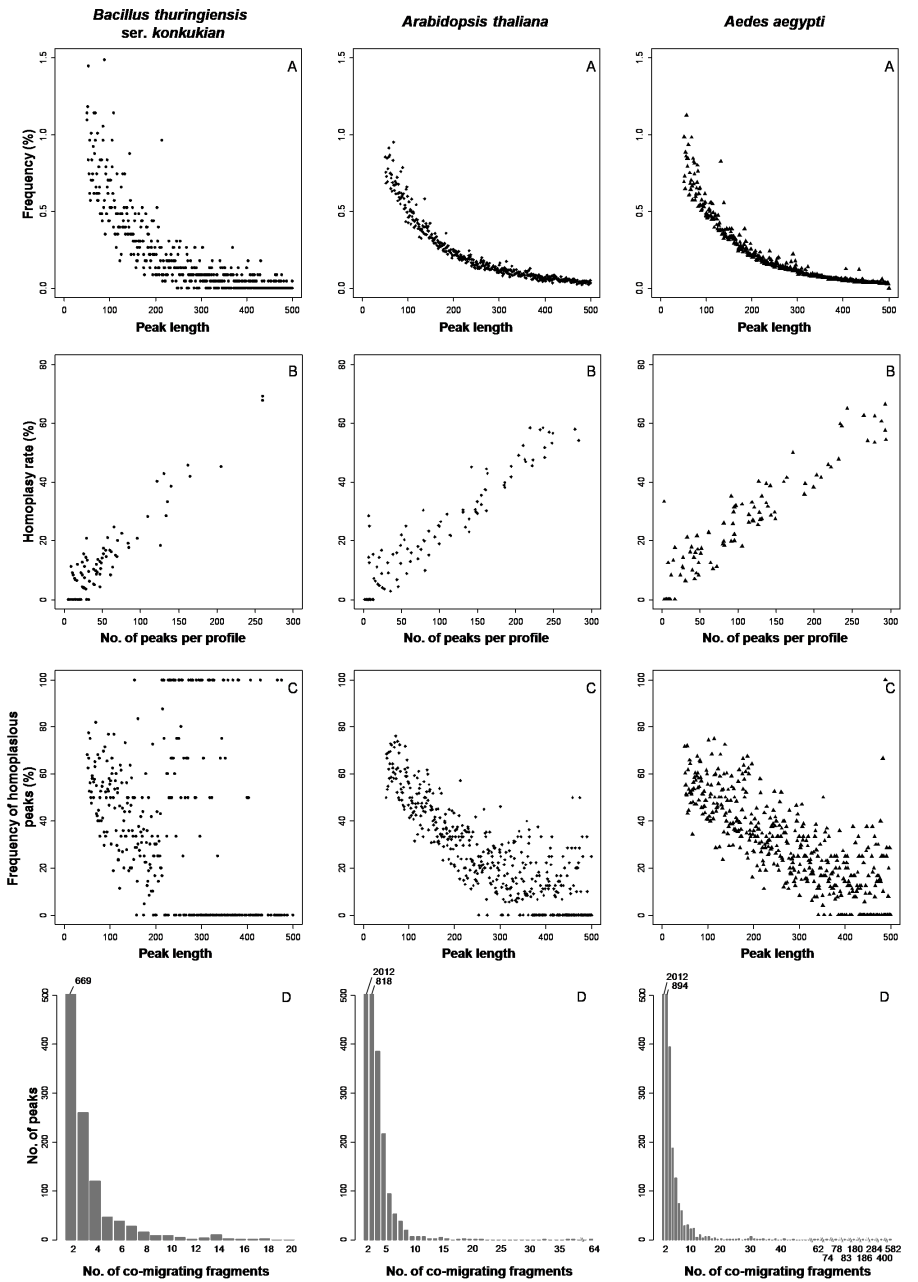
***In silico *AFLP results obtained using 84, 100 and 100 primer combinations for the species *Bacillus thuringiensis *ser. *konkukian, Arabidopsis thaliana *and *Aedes aegypti*, respectively**. A) Length distribution of the number of AFLP peaks obtained between 50 and 500 bp. B) Relationship between the homoplasy rate and the number of peaks per AFLP profile. C) Relationship between the frequency of homoplasic peaks and fragment length. D) Distribution of the number of co-migrating fragments in peaks (Y axis truncated at 80, real numbers indicated above each bar).

*In silico *analyses generated a total of 5,345 fragments and 2,709 peaks for the model species *B. thuringiensis *ser. *konkukian *(5 to 260 peaks per primer combination using 1 to 3 selective bases), 17,425 fragments and 9,907 peaks for *A. thaliana *(2 to 283 peaks per primer combination using 4 to 6 selective bases), and 21,729 fragments and 10,138 peaks for *Ae. aegypti *(3 to 294 peaks per primer combination using 5 to 8 selective bases). For each species, the mean number of peaks per group of primer combination with a similar number of selective bases (1 to 8 when considering the total number of selective bases added for the 2 primers) and nucleotide composition (GC content) is shown in Table 1. The results of all 284 primer combinations are presented in Additional file 1. The total peak length distribution (i.e. without selective bases) showed that the number of small length peaks greatly exceeded that of longer length peaks for the three genomes (Figure 2A). Fragment length distribution did not differ between the eukaryote and prokaryote genomes (Table 2).

**Table 1:** Summary of the number of peaks per profile, homoplasy rate and maximum number of co-migrating fragments obtained *in silico *with different numbers of selective bases for each species.

	Primer combinations			No. of peaks		Homoplasy rate (%)		
Species	No. of sb^*a*^	GC cont^*b*^	No tested	Mean	Range	Mean	Range	Max fragm^*c*^
*Bacillus thuringiensis *ser. *Konkukian*	1	AT	2	260	260 - 260	68.5	67.7 - 69.2	18
		GC	2	184	162 - 206	45.4	45.2 - 45.7	18
	2	AT	4	140	122 - 165	37.2	28.4 - 41.8	16
		m	8	98	47 - 136	22.8	8.5 - 42.8	6
		GC	4	60	42 - 76	13.9	8.2 - 22.3	14
	3	AT	32	46	18 - 96	8.9	0 - 24.6	14
		GC	32	21	5 - 47	7.8	0 - 15.4	14
								
*Arabidopsis thaliana*	4	AT	27	207	139 - 283	46.6	27.3 - 58.5	64
		m	17	129	56 - 185	29.3	21.9 - 45.1	21
		GC	9	62	28 - 94	13.3	3.6 - 21.3	5
	5	AT	12	77	32 - 115	18.5	8.8 - 30.4	8
		GC	9	35	18 - 50	12.1	2.8 - 22	38
	6	AT	5	26	12 - 54	8.8	0 - 20.4	3
		m	11	12	4 - 21	4.3	0 - 15.4	3
		GC	10	7	2 - 12	6.6	0 - 28.5	33
								
*Aedes aegypti*	5	AT	6	266	232 - 294	58.4	47.8 - 66.6	61
		GC	8	253	209 - 293	53.2	41.6 - 62.4	582
	6	AT	17	156	94 - 234	36.7	27.5 - 59.8	400
		m	22	109	61 - 148	27.8	18.1 - 40.2	284
		GC	14	58	33 - 97	15.8	7 - 32	180
	7	AT	8	44	27 - 62	13.6	6.5 - 22.6	7
		GC	5	25	17 - 39	11.6	0 - 18	6
	8	AT	10	10	3 - 28	7.0	0 - 33.3	2
		m	5	8	5 - 12	1.7	0 - 8.3	3
		GC	5	6	3 - 11	6.7	0 - 33.3	2

^*a *^Total number of selective bases added for the 2 primers.

^*b *^"GC cont" is composed of three classes of selective bases differing according to the proportion of their GC content: "AT" corresponds to selective bases containing a larger number of A or T, "GC" to selective bases containing a larger number of C or G, "m" to selective bases containing same number of A or T and C or G.

^*c *^Maximum number of co-migrating fragments in one single peak.

**Table 2:** Summary of generalized linear model results. Each row corresponds to a single model using multiple explanatory variables. Significant values are indicated in bold. Quasibinomial and negative-binomial error distributions were used and results are given after calculating type-II analysis-of-variance using a *F *test (Quasibinomial family) or a likelihood ratio test (negative-binomial family).

		Explanatory variables					
		
		Upstream parameters	AFLP parameters		AFLP diagnostics		
				
		Species	No. of sb^a^	CG cont	No. of peaks per profile	Peak length	Homoplasy per peak
Response variables	Distribution	(Df = 2)	(Df = 1)	(Df = 2)	(Df = 1)	(Df = 1)	(Df = 1)
***In silico *analyses**							
Peak length distribution^b^	Quasibinomial	*F*_1,1349 _= 2.09e-12, *P = 1*				*F*_1,1349 _= 967.20, ***P < 0.001***	
No. of peaks per profile	Negative binomial	χ^2 ^= 1574.87, ***P < 0.001***	χ^2 ^= 1869.47, ***P *< 0.001**	χ^2 ^= 275.24, ***P *< 0.001**			
Homoplasy rate H	Quasibinomial	*F*_2,278 _= 0.98, *P = 0.38*		*F*_2,278 _= 0.72, *P = 0.49*	*F*_1,278 _= 576.26, ***P *<*0.001***		
No. of CF^c^	Negative binomial	χ^2 ^= 19.90, ***P < 0.001***		χ^2 ^= 2.99, *P = 0.22*	χ^2 ^= 25.32, ***P < 0.001***	χ^2 ^= 37.92, ***P < 0.001***	
No. of CF^c ^in peaks with more than 10 fragments	Negative binomial	χ^2 ^= 6.96, ***P = 0.03***		χ^2 ^= 0.90, *P = 0.64*	χ^2 ^= 0.87, *P = 0.35*	χ^2 ^= 2.74, *P = 0.10*	
**Empirical analyses**							
Fluorescence intensity	Negative binomial					χ^2 ^= 22.33, ***P < 0.001***	χ^2 ^= 2.39, *P = 0.12*

^*a *^Total number of selective bases added for the 2 primers.

^*b *^Peak length distribution is expressed in relative frequencies.

^*c *^"CF" corresponds to comigrating fragments within a peak.

We first examined the effect of upstream parameters: genome features (size and repeated elements prevalence) and AFLP parameters (number of selective bases, GC content) on homoplasy. Homoplasy was measured using two statistics: the homoplasy rate (H) in each AFLP profile (i.e. ratio of the number of peaks containing co-migrating non-homologous fragments to the total number of peaks), and the number of co-migrating fragments per homoplasious peak. Then, we looked at the effect of these parameters on AFLP diagnostics (number of peaks and peak length distribution) to finally evaluate the accuracy of AFLP diagnostics to predict homoplasy.

### Effects of upstream parameters on homoplasy

Homoplasy rate calculated for each primer combination ranged from 0 to 69.2% for *B. thurigiensis *ser. *konkukian*, 0 to 58.5% for the model species *A. thaliana *and 0 to 66.6% for *Ae. aegypti *(Figure 2B). There was no significant effect of genome size on homoplasy rate, but we observed significant differences of the number of co-migrating fragments within a peak among the three species with different genome size (Table 2). Homoplasious peaks contained on average 3 co-migrating fragments for the model species *B. thurigiensis *ser. *konkukian *(range: 2 - 18) and *A. thaliana *(range: 2 - 64) and 4 co-migrating fragments for *Ae. aegypti *(range: 2 - 582). The presence of repetitive elements had a positive effect on the number of co-migrating fragments. A large frequency of co-migrating fragments was found in *Ae. aegypti *peaks (Figure 2D).

In our study, a maximum of 582 co-migrating fragments in one single peak was observed for *Ae. aegypti*. Of the 582 co-migrating fragments of 324 bp, 580 exhibited high similarity in sequence (mean identity index = 0.97, range: 0.84 - 1, calculated with Bioedit version 7.0.5 [39]) and corresponded to highly repetitive sequences in the genome. Using RepeatMasker (http://www.repeatmasker.org, [40]), the sequence was identified as a LINE retroelement. For *A. thaliana*, a maximum of 64 co-migrating fragments of 108 bp including 61 similar sequences (mean identity index = 0.97, range: 0.87 - 1) was observed, corresponding to a LTR element (Gypsy). For *B. thurigiensis *ser. *konkukian*, two peaks contained 18 co-migrating fragments of 89 and 144 bp and were both composed of 14 fragments with the same sequence (mean Identity index = 1) but located at different places in the genome. These sequences were not identified as transposable elements by RepeatMasker.

### Effects of upstream parameters on AFLP diagnostics

The number of selective bases determined the number of peaks generated and should be chosen according to the genome size under analysis; a larger number of selective bases reduced the number of fragments and peaks generated (Table 1). Furthermore, for the three species and for the same number of selective bases, the number of peaks generated decreased whilst the GC content of the selective bases increased (Table 2). For example, for *Ae. aegypti*, the primer combinations with 6 selective bases biased in AT produced more than twice the number of peaks than those biased in GC (mean = 156 and mean = 58 respectively, Table 1). The same trend was observed for the other species. The composition of primer combinations (i.e. GC content) had no significant effect on homoplasy rate and on the number of co-migrating fragments present in the peaks (Table 2).

### Accuracy of AFLP diagnostics to evaluate homoplasy

Generalized linear model showed a very strong effect of the number of peaks in a profile on the homoplasy rate (Table 2). Indeed, for all species a strong positive correlation was found between the number of peaks detected in a profile and the homoplasy rate (Figure 2B, Pearson's correlation: N = 84, r = 0.94, *P *< 0.001 for *B. thurigiensis *ser. *konkukian*; N = 100, r = 0.94, *P *< 0.001 for *A. thaliana *and N = 100, r = 0.93, *P *< 0.001 for *Ae. aegypti*). For example, in profiles with 100 peaks, about a quarter of peaks were composed of co-migrating fragments. The homoplasy rate decreased to around 15% for profiles with 50 peaks. In our study, only 43 profiles out of 284 did not present any co-migrating fragments but they contained few peaks (2 - 32 peaks). However, the choice of primer combinations producing less than 30 peaks does not guarantee the absence or a low level of homoplasy. For example, in the *A. thaliana*, the primer combination E+ACG/M+CTC generated a homoplasy rate of 28.5% in a profile with 40 AFLP fragments distributed in only 7 peaks.

The probability of peaks being homoplasious was negatively correlated with their length, (Figure 2C; *B. thurigiensis *ser. *Konkukian*: Pearson's correlation, r = -0.30, N = 329, *P *< 0.001; *A. thaliana*: r = -0.83, N = 451, *P *< 0.001 and *Ae. Aegypti*: r = -0.77, N = 451, *P *< 0.001), small length peaks more often contained co-migrating fragments. For example, peaks smaller than 100 bp in length, accounted for 36% of the homoplasy in our data. Our *in silico *results are in concordance with the theoretical finding that small peaks are more often homoplasious [18].

There was a significant positive effect of the total number of peaks in a profile on the number of co-migrating fragments present in peaks and a significant negative effect of peak length (Table 2). Indeed, homoplasious peaks contained more co-migrating fragments in profiles with a large number of peaks and in a smaller length range. However, when considering only peaks containing more than 10 co-migrating fragments (183 peaks), no more effect of peak size or effect of the number of peaks in a profile on the number of co-migrating fragments were detected (Table 2). This suggests a random size distribution of peaks containing a large number of fragments.

### Detecting homoplasious peaks in empirical profiles

A total of 118 peaks were generated by the seven primer combinations on the model species *Arabidopsis thaliana*, of which 10 were homoplasious. There was a significant effect of peak size on peak fluorescence intensity and no significant effect of homoplasy (Table 2). Homoplasious peaks tended to exhibit higher fluorescence intensity than non-homoplasious peaks (means ± sd: 6803 ± 3632 rfu and 4198 ± 3303 rfu respectively), but many non-homoplasious peaks also exhibited high fluorescence, so that peak intensity may not represent a valuable quality criterion in detecting homoplasious peaks.

### Predicting the AFLP profiles of non-model species

**Figure 3:**
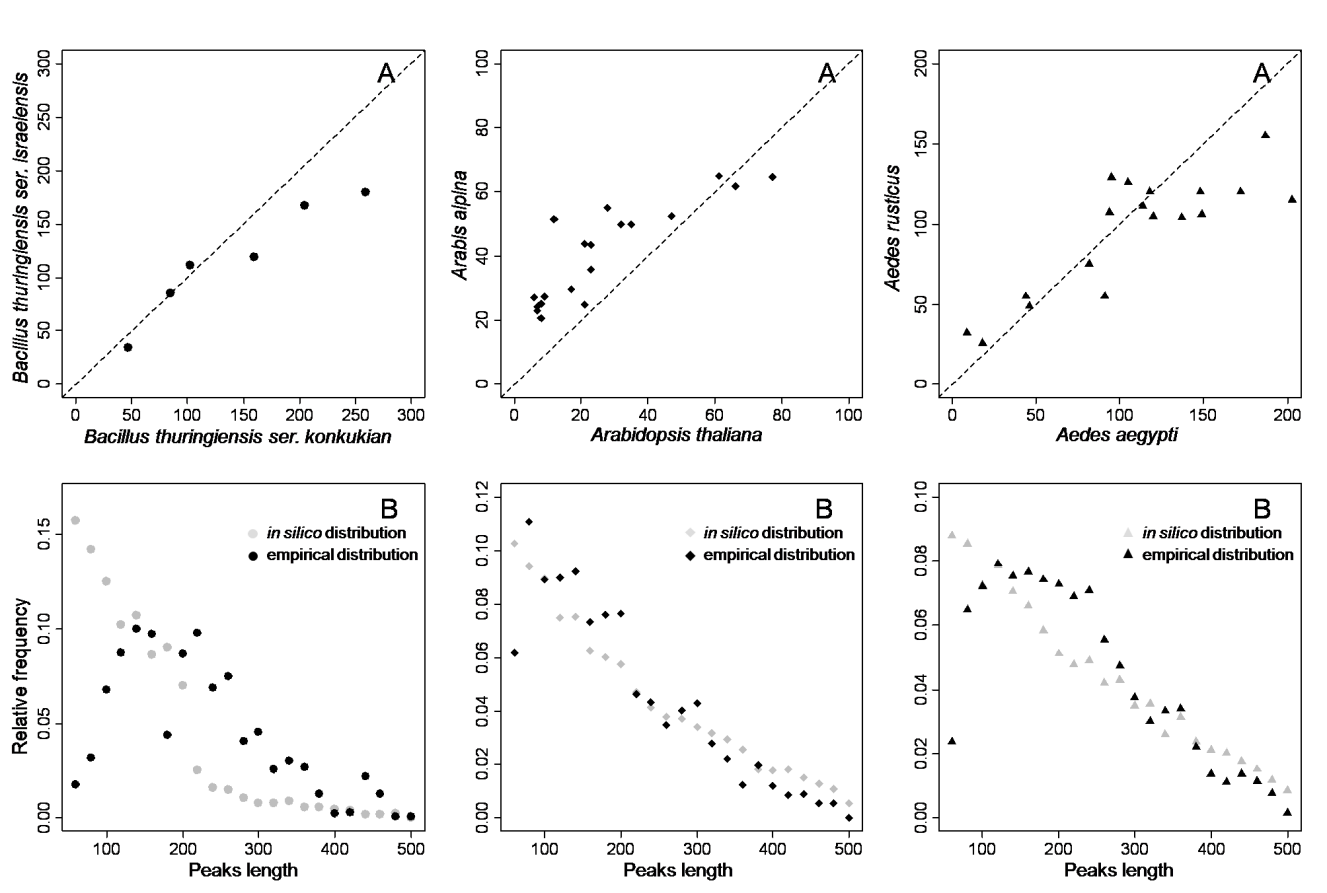
***In silico *AFLPs obtained on model species *vs*. empirical AFLPs obtained on related non-model species**. A) Comparison between numbers of peaks per profile obtained for model and non-model species. B) AFLP peak length distribution obtained *in silico *and empirically. Peaks were grouped by classes of 20 bp-length. Symbols ● corresponded to bacteria species, ◆ to plant species and ▲ to insect species.

The number of fragments obtained *in silico *for three model species, the bacterium *B. thuringiensis *ser. *konkukian*, the plant *A. thaliana *and the insect *Ae. aegypti*, was compared with that obtained for closely related species, *B. thuringiensis *ser. *israelensis*, *Arabis alpina *and *Aedes rusticus*, for which no full genome sequences were available (Figure 3A). The details for each model/non-model species pair and each primer combination are presented in Additional file 2.

For all species pairs, we found a positive, significant relationship between the number of *in silico *fragments and the average number of experimental fragments (Pearson's correlation: N = 6, r = 0.96, *P *= 0.003 for bacteria; N = 19, r = 0.86, *P *< 0.001 for plants and N = 20, r = 0.86, *P *< 0.001 for mosquitoes). The number of fragments in *A. alpina *tended to be larger than the number of *in silico *fragments of *A. thaliana*. This difference was not surprising since the *A. alpina *genome (2n = 16) is larger than the *A. thaliana *genome (2n = 10, [41]). For *Bacillus *and *Aedes*, the relationship between model and non-model species was close to a linear function with slope 1 and intercept 0 for profiles with less than 150 peaks (Figure 3A). However *in silico *AFLP tended to over-estimate the number of peaks in non-model species for primer combinations generating more than 150 peaks per profile (Figure 3A).

The distribution of peak length differed for all model/non-model species pairs. For all non-model species, small peaks (less than 70-100 bp depending on species) were significantly below the number expected *in silico *in model species (Figure 3B). For mosquitoes and plants, large peaks (more than 300 bp) were also overestimated *in silico*. This pattern was not present in *Bacillus*, probably because only few peaks are predicted in this range.

## Discussion

### Lessons of *in silico *AFLPs to detect homoplasy

The ISIF program allows to accurately predict AFLP profiles in model species using the genomic information available. The correspondence between *in silico *and empirical AFLP profiles has already been described for simple model species of bacteria [42,43] and for the plant *A. thaliana *[34].

The experimental AFLP procedure generated the peaks predicted by the *in silico *analysis carried out using ISIF, except for one long fragment (> 400 bp) insufficiently amplified to be detectable on the electrophoresis profile. However, this fragment was detected by pyrosequencing. The decrease in AFLP peak intensity as peak length increases is a well known phenomenon [28,44] illustrated here (see Figure 1). In our profile, fluorescence showed a decreasing intensity in high fragment lengths, except for the 318 bp peak containing 6 co-migrating fragments which exhibited much higher fluorescence intensity than the peaks of similar length. This suggests that fluorescence intensity could be a good indicator for peaks containing many different co-migrating fragments. However, detecting homoplasious peaks in an experimental AFLP profile based on fluorescence intensity remains a challenge, because the intensity of a peak does not necessarily reflect the actual number of different co-migrating fragments. For example, in the profile shown in Figure 1, the 113 bp peak contains two co-migrating fragments, but is less intense than the 115 bp peak which is not homoplasious. Overall, in *A. thaliana*, the effect of homoplasy on peak fluorescence intensity was not significant (Table 2). This limitation in detecting *a posteriori *homoplasious peaks in an experimental profile highlights the need to develop tools to limit *a priori *the probability of generating homoplasious peaks. *In silico *AFLPs make it possible to choose the best primer combinations prior to carrying out the experimental procedure.

### Homoplasy in model species

A high level of homoplasy was found when analyzing all study species, ranging up to 69%. For all species, homoplasy was highly dependent on the number of peaks generated per profile. It was about 15% in profiles containing 50 peaks and as much as 25% in profiles containing 100 peaks. This is in the range of homoplasy theoretically predicted by modeling the distribution of fragment lengths and calculating homoplasy rates given the fragment number in a profile [25], or experimentally estimated in sugar beet (13%) [24]. Given that the aim of most AFLP studies is to generate a large number of polymorphic markers at the lowest cost, many AFLP based studies are likely to contain a large number of homoplasious peaks.

For a given number of selective bases, the number of peaks per profile depends on genome size. To obtain a 50-peaks profile, a total of 3 selective bases are needed for *B. thuringiensis var konkukian*, 5 - 6 for *A. thaliana*, and 6 - 7 for *Ae. aegypti*. Accordingly, Altholff et al. [13] obtained 0 to 189 peaks per profile when carrying out *in silico *AFLP with 6 selective bases primer combinations of 8 taxa with genome size ranging from 5.23 to 2900 Mb. In their study, homoplasy ranged from 0% for bacteria with profiles containing 0 or 1 peak to 49% for a human profile containing 171 peaks. The authors concluded that homoplasy is dependant on genome size. However, by using the same primer combinations for all species, they could not distinguish between the linked effects of genome size and of the number of peaks per profile. In our study, a large range of primer combinations adapted for each species was used to obtain profiles containing at least 5 to 260 peaks per species, allowing for discrimination between the effects of genome size and the number of peaks. For a given number of peaks per profile, the homoplasy rate (i.e. the proportion of peaks containing co-migrating fragments) was apparently not related to genome size or systematic position.

However, homoplasious peaks can contain a larger number of co-migrating fragments in large and complex genomes. Furthermore, even if small length peaks are more likely to be homoplasious than longer peaks as previously reported [12], our results suggest that this can be not valid for peaks containing more than 10 fragments, as we observed highly homoplasious peaks at almost any length. The most frequent type of homoplasious peak is made up of two or more fragments of different sequences, co-migrating by chance. Small peaks are more likely to belong to this type, because of the skewed fragment length distribution (Figure 2A). Less frequently, homoplasious peaks are made up of many highly repetitive sequences. This is more likely to be found in large genomes, as they usually contain many repetitive sequences, such as transposable elements [37,45]. For example, an impressive 582 co-migrating, highly similar fragments was found in *Ae. aegypti*. This is not completely unexpected as 47% of the *Ae. aegypti *genome consists of transposable elements which can exhibit up to 50,000 copies per genome [38,46]. A strategy that could be used to avoid cutting in transposable elements is to use restriction enzymes sensitive to DNA methylation, because many transposable elements are known to be silenced by methylation [47]. However, this strategy cannot be routinely recommended to AFLP users, because transposable elements silencing is not only species dependent, but also tissue-dependent.

All these results highlight the importance of the choice of primer combination for the quality and the practical usefulness of the AFLP profiles. Both the number and the GC content of selective bases can have a strong effect on the number of peaks per profile, and therefore on homoplasy. In our case, the three genomes analyzed are AT-rich, so that primers with AT selective bases generate more peaks and therefore more homoplasious profiles. Furthermore, some combinations generate a particularly high homoplasy rate, or amplify repeated sequences.

### Predicting the AFLP profiles of non-model species

The *in silico *AFLP profile obtained from the model species can help to predict the AFLP profiles obtained in related species, for which complete genomic information does not exist. For the three pairs of species analysed, we observed a strong correlation between the predicted and observed profiles. The correlation was particularly strong between two varieties of the same species, *Bacillus thuringiensis *ser. *konkukian *and var *israelensis*, but remained very strong between species of the same genus (*Aedes*) and even between two species from different genera (*Arabidopsis *and *Arabis*) and with different genome sizes (two-fold difference [41]). In model species, the homoplasy rate is strongly correlated to the profile quality (number and size distribution of peaks generated). As non-model species profile quality is correlated to that obtained in model species, the extent of homoplasy in non-model species can be predicted from their profile quality.

*In silico *AFLPs tended to over-estimate the number of peaks expected in non-model species for profiles with more than 150 peaks. This phenomenon has already been described in the tetraploid species *Damasonium alisma *that produced less AFLP peaks using a primer combination with 6 selective bases than using a primer combination with the same 6 selective bases plus an additional A, T, G or C [29]. For the authors, many of the loci were insufficiently amplified using the 6 selective bases primer combination to produce peaks higher than the scoring threshold and were therefore not scored. Indeed, the decrease in the number of AFLP peaks scored in profiles with a large number of peaks may be due to multiple causes, including scoring errors in complex profiles [48,49], competition across fragments during the amplification process [29], and the poor amplification of longer fragments [28,44]. According to this later hypothesis, we observed less large fragments (more than 300 bp) than expected in all our *in silico*/experimental comparisons, except for the *Bacillus *pair of species for which few large fragments were expected. Furthermore, less small fragments than expected were observed in all our *in silico*/experimental comparisons. This could be due to the loss of small fragments during the purification step before separation, although the purification protocol we used is supposed to retain fragments larger than 30 bp; or they could be lost during the electrophoresis separation.

Finally, the quality of sequences in published model species genomes could be a further source of bias in the number of peaks predicted with *in silico *AFLP. Indeed, most available model genomes contain genotyping errors or gaps represented by a series of Ns, even for the model species *A. thaliana*. Finally, the fragmentation of most large published genomes into thousands of scaffolds (for example, the *Ae. aegypti *genome is composed of 4,768 supercontigs) may further bias the estimation of the number of fragments amplified.

### Recommendations

The optimization of the AFLP reaction (and especially the choice of selective primers) is often achieved through an empirical procedure. For instance, the polymorphism and the reproducibility of markers are generally the most important criteria on which the choice of AFLP primers is based. However, such optimization procedures are probably not able to avoid, in a reliable way, the occurrence of size homoplasy in AFLP profiles. Here we show that ISIF allows a rapid screen of candidate restriction enzymes and/or combinations of selective bases during the optimization steps of the AFLP reaction. Low level of homoplasy at the intra-individual level is likely to coincide with low level of homoplasy at the inter-individual level. *In silico *AFLPs can be used to prevent homoplasy in AFLP data sets and in turn, reduce biases in population genetics, conservation of genetic resources or genome scan analyses. In addition, the present study shows that genomic sequences of model species can be used to predict AFLP profiles generated in related non-model species. Finally, ISIF represents a key tool to plan the number of fragments to be sequenced in complex and costly high throughout genomic experiments such as pyrosequencing, or to address further questions such as evaluating the distribution of restriction fragments in genomes. We conclude the present study with general suggestions on the choice of primer combinations and the process for peak selection. These suggestions in addition to recommendations made in Gort et al. [18] will help to improve experimental AFLP studies on both model and non-model species.

• Primer combinations generating less than 30 AFLP peaks per profile can help to limit homoplasy within a profile. In practice, the total number of six selective bases originally recommended by Vos et al. [5], generally used in most published studies, is insufficient in preventing homoplasy in most plant and animal genomes. In addition, the choice of selective bases biased in GC content can reduce markedly the number of peaks generated for many species. Furthermore, the choice of restriction enzymes with high GC content recognition sites is likely to reduce the number of restriction fragments for low GC content genomes.

• If possible, primer combinations which amplify repetitive sequences should be avoided.

• Peaks smaller than 100 bp in length can pose problems, as they can be responsible of more than one third of homoplasy. Our analysis suggests that focusing on the longer fragments would help reduce this issue.

• Peaks of large length that exhibit particularly high fluorescence intensity compared to peaks of a similar length in the same profile should be considered with caution, as they often contain several non-homologous co-migrating fragments.

• The mean number of peaks per profile should always be mentioned in AFLP studies to allow the evaluation of the homoplasy rate within profiles. Indeed, most of the published studies only mention the total number of polymorphic peaks scored (but see Meyer et al. [50]). However, this does not correspond to the number of peaks per profile, which depends on the relatedness across individuals and on the number of individuals genotyped. For example, the analysis of a small number of individuals, of related individuals or of poorly differentiated populations can lead to the identification of only a small total number of polymorphic fragments, but with a large number of fragments per profile.

## Conclusions

Carrying out *in silico *analyses before the experimental work allows a rapid screen of candidate restriction enzymes and the combinations of selective bases to be used, in order to optimize the experimental work. It can also help to plan the number of fragments to be sequenced in complex and costly high throughput genomic experiments such as pyrosequencing. Most importantly, *in silico *AFLP can help to limit homoplasy in AFLP data sets, reducing biases in population genetics, conservation of genetic resources or genome scan analyses. Finally, *in silico *analysis represents a key tool to address further questions such as evaluating the distribution of restriction fragments in genomes.

## Methods

### *In silico *AFLPs on model species

Three model species for which the full genome sequences are available were used in this study: *Bacillus thuringiensis *ser. *konkukian, Arabidopsis thaliana *(ecotype Columbia) and *Aedes aegypti *(Liverpool strain). *Bacillus thuringiensis *ser. *konkukian *genome and plasmid were obtained from GenBank (accession number AE017355 and CP000047), *Arabidopsis thaliana *genome was obtained from The Arabidopsis Information Resource http://www.arabidopsis.org, and *Aedes aegypti *genome was obtained from VectorBase http://www.vectorbase.org. *In silico *AFLPs were performed on total genomic DNA for *A. thaliana *and *Ae. aegypti *without taking into account organellar genomes that are negligible in length in comparison to the nuclear genome [13]. For bacteria the length of the plasmidic genome is of significant length in the whole genome, therefore both genomic DNA and plasmid DNA where used for *B. thuringiensis *ser. *konkukian*. For all *in silico *analyses, fragments between 50 and 500 pb were considered.

For *B. thuringiensis *ser. *Konkukian, in silico *AFLP profiles were generated using all possible combinations of primers EcoRI/MseI: E+0/M+1, E+1/M+1 and E+1/M+2 (84 combinations). For the species *A. thaliana *and *Ae. aegypti*, 100 EcoRI/MseI primer combinations were randomly chosen among combinations containing between 3 to 5 selective bases and 5 to 8 selective bases respectively (Additional file 1). For each species, the number and the sequence of the selective bases used for amplification were chosen in order to generate less than 300 peaks per profile. Producing profiles with more than 200 peaks is unrealistic in practice but such situations were chosen here to ensure a high homoplasy rate. For each *in silico *profile, the number of non-identical fragments (i.e. fragments with different sequences or chromosomal positions) and the number of detectable peaks (i.e. regrouping all co-migrating fragments) were determined. All statistical analyses were carried out using R software version 2.5 [51].

We tested the effects of the number of peaks in profiles and of species identity on H in a generalized linear model (GLM) with a Quasibinomial error. Subsequently, for each species we performed Pearson's correlation between H and the number of peaks per profile, and between H and peak length. The effects of the number of peaks in profiles, peak length, species identity and composition of selective bases (i.e. GC content) on the number of co-migrating fragments in homoplasious peaks were tested in a GLM; models with Poisson error distribution showed evidence of overdispersion, therefore we used a negative -binomial family to build models [52]. The effects of these four parameters on the number of co-migrating fragments in homoplasious peaks were then tested in a GLM including only peaks containing more than 10 co-migrating fragments. For GLMs, significance was calculated using type-II analysis-of-variance using a *F *test (quasibinomial models) or a likelihood ratio test (negative-binomial models) [52].

### Empirical AFLP profiles in a model species

The genome of the model species *Arabidopsis thaliana *was used to validate the reliability of both experimental AFLP and of the results of ISIF *in silico *AFLP. The advantage of using this selfing plant is that the published genome (Ecotype Columbia) is identical to the genome of all plants from this ecotype. An *in silico *profile using the primer combination EcoRI+ATG/MseI+ATG was generated and compared with the corresponding experimental AFLP profile. This primer combination was chosen because *in silico *it generated two homoplasious peaks (containing 2 and 6 co-migrating fragments, respectively). AFLP analyses were obtained according to Paris et al. [53]. In short, 150 ng of the total genomic DNA was first digested with 2 units of EcoRI (New England Biolabs) for 2.5 hours at 37°C, and then with 5 units of MseI (New England Biolabs) in the same conditions. Specific oligonucleotide adaptors were then ligated to the end of the restriction fragments with 1 unit of T4 DNA ligase (New England Biolabs) for 3 hours at 37°C. Pre-selective and selective amplifications were performed with 0.2 μM of primers complementary to the adaptor sequences after 20 times dilution of the digestion/ligation product and 10 times dilution of the pre-selective PCR product. Labeled selective fragments were separated by electrophoresis on an ABI 3130 capillary sequencer (Applied Biosystems). AFLP patterns were then visualized with GeneMapper V3.7 software (Applied Biosystems): a fluorescent peak corresponds to the presence of an amplified restriction fragment. A scoring threshold of 500 rfu in fluorescence intensity was set up to detect peaks. For each sample, all peaks between 50 and 500 pb were considered. Reproducibility of the AFLP method was checked by carrying out the whole AFLP protocol three times as recommended by Bonin et al. [48]. All peaks of the experimental AFLP profile were sequenced to confirm homology among experimental and *in silico *fragments both in length and in sequence using pyrosequencing 454 Life Science and the GS 20 protocol (Roche Applied Science) following the manufacturer's instructions.

In order to determine if homoplasious peaks can be detected using their fluorescence intensity, we used 7 primer combinations (E+ATG/M+ATG, E+GC/M+GC, E+AAT/M+CAC, E+ATG/M+CTC, E+AGG/M+CAC, E+ATG/M+CAA, E+AGG/M+CAA) chosen because they produce *in silico *homoplasious peaks. A GLM with a negative -binomial family model was then used to test the effects of peak size and of homoplasy on peak intensity.

### Predictions of *in silico *AFLPs to non-model species

For the comparison between model and non-model species, the *in silico *AFLP profile prediction for model species was performed on 6 primer combinations EcoRI/MseI for *B. thuringiensis *ser. *konkukian*, 7 primer combinations EcoRI/MseI and 12 primer combinations PstI/MseI for *A. thaliana*, and 20 primer combinations EcoRI/MseI for *Ae. aegypti *(Additional file 2). Three non-model species were used for these analyses: the bacterium *Bacillus thuringiensis *ser. *israelensis *of worldwide origin [54], the plant *Arabis alpina *collected from the Alps (France and Switzerland), and the mosquito *Aedes rusticus *collected in the Rhône-Alps region (France). For *B. thuringiensis *ser. *israelensis*, total genomic DNA was extracted from overnight culture at 27°C of isolated bacterial strains using the DNeasy tissue Kit (Qiagen) following the Gram positive bacteria protocol. Total genomic DNA was extracted from leaves of *A. alpina *using the DNeasy Plant Kit (Qiagen) and from larvae of *Ae. rusticus *and using the DNeasy tissue Kit (Qiagen), according to the manufacturer's instructions.

All the experimental AFLP profiles were generated using the protocol described above, and using the same primer combinations described in the *in silico *analyses. Finally, experimental profiles were generated on 2 - 23 individuals depending on the primer combinationfor *B. thuringiensis *ser. *israelensis*, on 123 - 728 *A. alpina *plants, and on 2 - 279 *Ae. rusticus *larvae. For each pair of model/non-model species, a Pearson's correlation between the number of AFLP fragments obtained *in silico *and experimental AFLP profiles was performed. We also compared the fragment length distribution for each pair of model/non model species using GLMs on peak frequencies. Peaks were categorized by groups of 20 bp-length, and a GLM was performed for each of the 23 groups, with 'model' or 'non-model' as fixed effect.

## Authors' contributions

MP conceived the overall study, carried out the *in silico *analysis on model species, produced the experimental data set for all species except *Arabis alpina*, analyzed the data set and drafted the manuscript. BB conceived the ISIF program, GFF helped with the analyses and helped draft the manuscript, BNP produced the *Arabis alpina *dataset and helped for data analysis. LD took part to the data analysis and wrote substantial parts of the manuscript. All authors read and approved the final manuscript.

## Supplementary Material

Additional file 1 Table S1. Summary of the number of fragments and peaks per profile, homoplasy rate and maximum number of co-migrating fragments obtained *in silico *with all 284 different EcoRI/MseI primer combination pairs. Click here for file

Additional file 2 Table S2. Primer combinations and sample sizes used for each model/non-model species comparison and number of in silico and empirical peaks obtained. Click here for file
