# Supplementary material for: Amplified fragment length homoplasy: in silico analysis for model and non-model species
Source: BMC Genomics. 2010 May 7;11:287. doi: 10.1186/1471-2164-11-287 (PMC2875239; doi:10.1186/1471-2164-11-287)
Supplement: Additional file 1 — Table S1. Summary of the number of fragments and peaks per profile, homoplasy rate and maximum number of co-migrating fragments obtained in silico with all 284 different EcoRI/MseI primer combination pairs. [file 1471-2164-11-287-S1.DOC]

Additional file 1 - Table S1. Summary of the number of fragments and peaks per profile, homoplasy rate and maximum number of co-migrating fragments obtained *in silico* with all 284 different EcoRI/MseI primer combination pairs.

| **Species** | **No. of** **sb***^a^* | **GC cont***^b^* | **EcoRI selective bases** | **MseI selective bases** | **No. of fragments** | **No. of peaks** | **Homoplasy rate (%)** | **Max fragm***^c^* |
| --- | --- | --- | --- | --- | --- | --- | --- | --- |
| *Bacillus thuringiensis* ser. *konkukian* | 1 | AT | - | A | 899 | 260 | 69.23 | 14 |
|  | 1 | AT | - | T | 833 | 260 | 67.69 | 18 |
|  | 1 | GC | - | C | 432 | 206 | 45.15 | 17 |
|  | 1 | GC | - | G | 309 | 162 | 45.68 | 18 |
|  | 2 | AT | A | A | 287 | 165 | 41.82 | 7 |
|  | 2 | AT | A | T | 243 | 140 | 38.57 | 16 |
|  | 2 | AT | T | A | 198 | 122 | 40.16 | 5 |
|  | 2 | AT | T | T | 189 | 134 | 28.36 | 5 |
|  | 2 | m | A | C | 107 | 85 | 17.65 | 4 |
|  | 2 | m | A | G | 54 | 47 | 8.51 | 4 |
|  | 2 | m | C | A | 217 | 131 | 42.75 | 5 |
|  | 2 | m | C | T | 203 | 136 | 33.09 | 6 |
|  | 2 | m | G | A | 158 | 110 | 28.18 | 4 |
|  | 2 | m | G | T | 159 | 126 | 18.25 | 4 |
|  | 2 | m | T | C | 104 | 84 | 19.05 | 3 |
|  | 2 | m | T | G | 79 | 67 | 14.93 | 3 |
|  | 2 | GC | C | C | 110 | 76 | 22.37 | 14 |
|  | 2 | GC | C | G | 76 | 61 | 8.2 | 14 |
|  | 2 | GC | G | C | 72 | 60 | 16.67 | 3 |
|  | 2 | GC | G | G | 58 | 42 | 8.52 | 14 |
|  | 3 | AT | A | AA | 127 | 96 | 20.83 | 6 |
|  | 3 | AT | A | AC | 35 | 33 | 6.06 | 2 |
|  | 3 | AT | A | AG | 60 | 52 | 15.38 | 2 |
|  | 3 | AT | A | AT | 71 | 57 | 21.05 | 3 |
|  | 3 | AT | A | CA | 49 | 42 | 11.9 | 3 |
|  | 3 | AT | A | CT | 24 | 22 | 9.09 | 2 |
|  | 3 | AT | A | GA | 18 | 18 | 0 | 1 |
|  | 3 | AT | A | GT | 20 | 20 | 0 | 1 |
|  | 3 | AT | A | TA | 93 | 69 | 14.59 | 14 |
|  | 3 | AT | A | TC | 29 | 28 | 3.47 | 2 |
|  | 3 | AT | A | TG | 63 | 52 | 17.31 | 3 |
|  | 3 | AT | A | TT | 64 | 55 | 14.54 | 2 |
|  | 3 | AT | C | AA | 85 | 70 | 20 | 3 |
|  | 3 | AT | C | AT | 47 | 43 | 9.3 | 2 |
|  | 3 | AT | C | TA | 56 | 49 | 12.24 | 3 |
|  | 3 | AT | C | TT | 77 | 62 | 16.13 | 6 |
|  | 3 | AT | G | AA | 54 | 48 | 10.42 | 3 |
|  | 3 | AT | G | AT | 58 | 50 | 14 | 3 |
|  | 3 | AT | G | TA | 50 | 44 | 13.64 | 2 |
|  | 3 | AT | G | TT | 54 | 48 | 10.42 | 3 |
|  | 3 | AT | T | AA | 83 | 65 | 24.62 | 3 |
|  | 3 | AT | T | AC | 32 | 32 | 0 | 1 |
|  | 3 | AT | T | AG | 41 | 39 | 5.13 | 2 |
|  | 3 | AT | T | AT | 63 | 54 | 16.67 | 2 |
|  | 3 | AT | T | CA | 45 | 41 | 9.76 | 2 |
|  | 3 | AT | T | CT | 34 | 30 | 13.33 | 2 |
|  | 3 | AT | T | GA | 29 | 27 | 7.41 | 2 |
|  | 3 | AT | T | GT | 28 | 27 | 3.7 | 2 |
|  | 3 | AT | T | TA | 54 | 48 | 10.42 | 3 |
|  | 3 | AT | T | TC | 35 | 29 | 20.69 | 2 |
|  | 3 | AT | T | TG | 49 | 47 | 4.26 | 2 |
|  | 3 | AT | T | TT | 72 | 64 | 10.94 | 3 |
|  | 3 | GC | A | CC | 10 | 9 | 11.1 | 2 |
|  | 3 | GC | A | CG | 30 | 27 | 11.11 | 2 |
|  | 3 | GC | A | GC | 13 | 11 | 9.09 | 3 |
|  | 3 | GC | A | GG | 9 | 9 | 0 | 1 |
|  | 3 | GC | C | AC | 35 | 32 | 9.38 | 2 |
|  | 3 | GC | C | AG | 53 | 47 | 12.77 | 2 |
|  | 3 | GC | C | CA | 42 | 40 | 5 | 2 |
|  | 3 | GC | C | CC | 6 | 6 | 0 | 1 |
|  | 3 | GC | C | CG | 27 | 24 | 12.5 | 2 |
|  | 3 | GC | C | CT | 38 | 24 | 8.33 | 14 |
|  | 3 | GC | C | GA | 26 | 25 | 4 | 2 |
|  | 3 | GC | C | GC | 15 | 14 | 7.14 | 2 |
|  | 3 | GC | C | GG | 12 | 12 | 0 | 1 |
|  | 3 | GC | C | GT | 29 | 16 | 6.25 | 14 |
|  | 3 | GC | C | TC | 30 | 26 | 15.38 | 2 |
|  | 3 | GC | C | TG | 43 | 39 | 10.26 | 2 |
|  | 3 | GC | G | AC | 27 | 26 | 3.85 | 2 |
|  | 3 | GC | G | AG | 25 | 24 | 4.17 | 2 |
|  | 3 | GC | G | CA | 32 | 28 | 14.29 | 2 |
|  | 3 | GC | G | CC | 12 | 11 | 9.09 | 2 |
|  | 3 | GC | G | CG | 18 | 18 | 0 | 1 |
|  | 3 | GC | G | CT | 16 | 15 | 6.67 | 2 |
|  | 3 | GC | G | GA | 15 | 15 | 0 | 1 |
|  | 3 | GC | G | GC | 13 | 12 | 8.33 | 2 |
|  | 3 | GC | G | GG | 5 | 5 | 0 | 1 |
|  | 3 | GC | G | GT | 31 | 17 | 11.76 | 14 |
|  | 3 | GC | G | TC | 29 | 29 | 0 | 1 |
|  | 3 | GC | G | TG | 32 | 32 | 0 | 1 |
|  | 3 | GC | T | CC | 17 | 17 | 0 | 1 |
|  | 3 | GC | T | CG | 29 | 27 | 7.41 | 2 |
|  | 3 | GC | T | GC | 22 | 22 | 0 | 1 |
|  | 3 | GC | T | GG | 21 | 21 | 0 | 1 |
| *Arabidopsis thaliana* | 4 | AT | AA | AC | 514 | 248 | 53.23 | 7 |
|  | 4 | AT | AA | AG | 582 | 249 | 56.63 | 22 |
|  | 4 | AT | AA | AT | 634 | 244 | 56.97 | 15 |
|  | 4 | AT | AA | CA | 529 | 238 | 51.68 | 14 |
|  | 4 | AT | AA | CT | 424 | 213 | 46.95 | 9 |
|  | 4 | AT | AA | TA | 527 | 236 | 58.47 | 9 |
|  | 4 | AT | AC | AA | 413 | 204 | 49.02 | 8 |
|  | 4 | AT | AT | AA | 729 | 278 | 57.91 | 37 |
|  | 4 | AT | AT | AT | 499 | 238 | 48.32 | 9 |
|  | 4 | AT | AT | CT | 293 | 186 | 38.17 | 6 |
|  | 4 | AT | AT | GA | 487 | 222 | 45.5 | 64 |
|  | 4 | AT | AT | TA | 402 | 194 | 45.36 | 11 |
|  | 4 | AT | AT | TG | 340 | 194 | 41.75 | 14 |
|  | 4 | AT | CT | AA | 328 | 186 | 39.78 | 12 |
|  | 4 | AT | CT | AT | 197 | 139 | 27.34 | 5 |
|  | 4 | AT | TA | AA | 531 | 219 | 58.45 | 11 |
|  | 4 | AT | TA | GA | 264 | 161 | 37.27 | 8 |
|  | 4 | AT | TA | GT | 251 | 150 | 33.33 | 10 |
|  | 4 | AT | TA | TA | 344 | 163 | 42.94 | 18 |
|  | 4 | AT | TA | TC | 273 | 162 | 44.44 | 7 |
|  | 4 | AT | TC | AA | 526 | 232 | 57.76 | 9 |
|  | 4 | AT | TG | AA | 403 | 210 | 52.38 | 8 |
|  | 4 | AT | TG | TA | 203 | 148 | 29.73 | 4 |
|  | 4 | AT | TG | TT | 248 | 155 | 35.48 | 8 |
|  | 4 | AT | TT | AA | 755 | 283 | 54.06 | 19 |
|  | 4 | AT | TT | TA | 391 | 212 | 47.64 | 6 |
|  | 4 | AT | TT | TT | 441 | 223 | 47.53 | 11 |
|  | 4 | m | AA | CG | 188 | 139 | 23.02 | 5 |
|  | 4 | m | AC | TG | 217 | 147 | 30.61 | 5 |
|  | 4 | m | AG | AG | 232 | 155 | 32.26 | 6 |
|  | 4 | m | AT | GC | 213 | 150 | 29.33 | 5 |
|  | 4 | m | CA | AC | 248 | 162 | 30.25 | 8 |
|  | 4 | m | CA | CA | 253 | 142 | 45.07 | 15 |
|  | 4 | m | CA | CT | 179 | 131 | 29.77 | 5 |
|  | 4 | m | CC | AT | 139 | 99 | 25.25 | 5 |
|  | 4 | m | CC | TA | 107 | 86 | 23.26 | 3 |
|  | 4 | m | CG | TA | 71 | 56 | 25 | 3 |
|  | 4 | m | CT | CT | 135 | 100 | 25 | 5 |
|  | 4 | m | CT | GA | 184 | 131 | 30.53 | 5 |
|  | 4 | m | GA | GA | 311 | 185 | 38.92 | 19 |
|  | 4 | m | GT | GT | 155 | 102 | 26.47 | 16 |
|  | 4 | m | GT | TC | 157 | 114 | 21.93 | 9 |
|  | 4 | m | TC | TC | 281 | 160 | 37.5 | 21 |
|  | 4 | m | TG | TG | 172 | 134 | 24.63 | 4 |
|  | 4 | GC | AC | GG | 118 | 94 | 21.28 | 5 |
|  | 4 | GC | CC | CA | 103 | 80 | 20 | 3 |
|  | 4 | GC | CG | CG | 29 | 28 | 3.57 | 2 |
|  | 4 | GC | CG | GA | 90 | 81 | 9.88 | 3 |
|  | 4 | GC | GC | CA | 82 | 72 | 13.89 | 2 |
|  | 4 | GC | GC | GC | 48 | 46 | 4.35 | 2 |
|  | 4 | GC | GC | GT | 5 | 49 | 12.24 | 2 |
|  | 4 | GC | GC | TC | 77 | 63 | 15.87 | 5 |
|  | 4 | GC | GT | CG | 63 | 49 | 18.37 | 5 |
|  | 5 | AT | AA | CGA | 89 | 72 | 18.06 | 4 |
|  | 5 | AT | AA | CGT | 67 | 57 | 8.77 | 5 |
|  | 5 | AT | AA | CTA | 130 | 100 | 19 | 5 |
|  | 5 | AT | AA | CTC | 116 | 79 | 30.38 | 8 |
|  | 5 | AT | AA | CTG | 102 | 79 | 20.25 | 5 |
|  | 5 | AT | AA | CTT | 154 | 110 | 29.09 | 6 |
|  | 5 | AT | AC | CTA | 37 | 32 | 12.5 | 3 |
|  | 5 | AT | AC | CTT | 70 | 62 | 12.9 | 2 |
|  | 5 | AT | AG | CTT | 68 | 58 | 17.24 | 2 |
|  | 5 | AT | AT | CAA | 146 | 115 | 21.74 | 4 |
|  | 5 | AT | AT | CTA | 89 | 76 | 17.11 | 2 |
|  | 5 | AT | AT | CTT | 103 | 86 | 15.12 | 4 |
|  | 5 | GC | AA | CGG | 62 | 50 | 22 | 3 |
|  | 5 | GC | AC | CTC | 83 | 44 | 6.82 | 38 |
|  | 5 | GC | AC | CTG | 26 | 24 | 8.83 | 2 |
|  | 5 | GC | AG | CGA | 28 | 24 | 16.69 | 2 |
|  | 5 | GC | AG | CGG | 20 | 18 | 11.11 | 2 |
|  | 5 | GC | AG | CGT | 38 | 33 | 9.09 | 3 |
|  | 5 | GC | AG | CTC | 52 | 44 | 15.91 | 3 |
|  | 5 | GC | AG | CTG | 64 | 39 | 15.38 | 20 |
|  | 5 | GC | AT | CGG | 39 | 36 | 2.78 | 4 |
|  | 6 | AT | AAT | CAC | 24 | 21 | 14.29 | 2 |
|  | 6 | AT | ATA | ATA | 66 | 54 | 20.37 | 3 |
|  | 6 | AT | ATG | CAA | 26 | 25 | 4 | 2 |
|  | 6 | AT | ATG | CAT | 20 | 19 | 5.26 | 2 |
|  | 6 | AT | ATG | CTA | 12 | 12 | 0 | 1 |
|  | 6 | m | ACA | CCA | 22 | 21 | 4.76 | 2 |
|  | 6 | m | AGG | CAA | 15 | 13 | 15.38 | 2 |
|  | 6 | m | AGG | CAT | 15 | 14 | 7.14 | 2 |
|  | 6 | m | AGG | CTA | 10 | 10 | 0 | 1 |
|  | 6 | m | ATC | CAC | 9 | 9 | 0 | 1 |
|  | 6 | m | ATG | CAC | 8 | 8 | 0 | 1 |
|  | 6 | m | ATG | CAG | 8 | 7 | 14.28 | 2 |
|  | 6 | m | ATG | CGA | 12 | 12 | 0 | 1 |
|  | 6 | m | ATG | CGT | 4 | 4 | 0 | 1 |
|  | 6 | m | ATG | CTC | 18 | 16 | 6.25 | 3 |
|  | 6 | m | ATG | CTG | 13 | 13 | 0 | 1 |
|  | 6 | GC | ACG | CAG | 7 | 7 | 0 | 1 |
|  | 6 | GC | ACG | CTC | 40 | 7 | 28.47 | 33 |
|  | 6 | GC | AGC | CAC | 7 | 7 | 0 | 1 |
|  | 6 | GC | AGC | CTG | 10 | 8 | 25 | 2 |
|  | 6 | GC | AGG | CAC | 9 | 8 | 12.5 | 2 |
|  | 6 | GC | AGG | CAG | 12 | 12 | 0 | 1 |
|  | 6 | GC | AGG | CGT | 6 | 6 | 0 | 1 |
|  | 6 | GC | AGG | CTC | 7 | 7 | 0 | 1 |
|  | 6 | GC | AGG | CTG | 5 | 5 | 0 | 1 |
|  | 6 | GC | ATG | CGC | 2 | 2 | 0 | 1 |
| Aedes aegypti | 5 | AT | AAT | CG | 668 | 265 | 62.64 | 14 |
|  | 5 | AT | AC | CTA | 636 | 243 | 65.14 | 37 |
|  | 5 | AT | AC | CTT | 732 | 270 | 54.07 | 61 |
|  | 5 | AT | AG | CTT | 713 | 294 | 54.42 | 21 |
|  | 5 | AT | AT | CCT | 995 | 293 | 66.55 | 42 |
|  | 5 | AT | ATA | CG | 477 | 232 | 47.84 | 18 |
|  | 5 | GC | AC | CTC | 649 | 222 | 45.05 | 186 |
|  | 5 | GC | AC | CTG | 637 | 279 | 53.41 | 35 |
|  | 5 | GC | AG | CGA | 1361 | 288 | 60.63 | 582 |
|  | 5 | GC | AG | CGG | 392 | 209 | 41.63 | 13 |
|  | 5 | GC | AG | CGT | 440 | 218 | 45.87 | 11 |
|  | 5 | GC | AG | CTC | 544 | 236 | 59.15 | 45 |
|  | 5 | GC | AG | CTG | 689 | 293 | 57.68 | 12 |
|  | 5 | GC | AT | CGG | 893 | 279 | 62.37 | 42 |
|  | 6 | AT | AAC | CAA | 407 | 172 | 50 | 27 |
|  | 6 | AT | AAT | CAA | 1143 | 234 | 59.83 | 400 |
|  | 6 | AT | AAT | CAC | 222 | 139 | 31.65 | 11 |
|  | 6 | AT | AAT | CTC | 217 | 128 | 29.69 | 11 |
|  | 6 | AT | AAT | CTG | 352 | 188 | 39.36 | 32 |
|  | 6 | AT | ACA | CAT | 244 | 121 | 31.4 | 62 |
|  | 6 | AT | ACT | CAA | 408 | 203 | 42.36 | 31 |
|  | 6 | AT | ATA | CAA | 309 | 187 | 35.83 | 7 |
|  | 6 | AT | ATA | CAC | 163 | 105 | 32.38 | 11 |
|  | 6 | AT | ATA | CTC | 158 | 94 | 29.79 | 11 |
|  | 6 | AT | ATA | CTG | 206 | 149 | 27.52 | 6 |
|  | 6 | AT | ATG | CAA | 338 | 199 | 38.19 | 7 |
|  | 6 | AT | ATG | CAT | 287 | 160 | 40 | 14 |
|  | 6 | AT | ATG | CTA | 194 | 131 | 27.48 | 6 |
|  | 6 | AT | ATG | CTT | 234 | 142 | 38.73 | 8 |
|  | 6 | AT | ATT | CCA | 230 | 143 | 28.67 | 15 |
|  | 6 | AT | ATT | CTA | 405 | 164 | 41.46 | 74 |
|  | 6 | m | AAC | CAC | 146 | 95 | 22.11 | 8 |
|  | 6 | m | AAC | CTC | 183 | 114 | 26.32 | 17 |
|  | 6 | m | AAC | CTG | 215 | 148 | 29.73 | 6 |
|  | 6 | m | ACA | CAG | 105 | 80 | 18.75 | 6 |
|  | 6 | m | ACG | CAA | 142 | 105 | 18.1 | 14 |
|  | 6 | m | ACT | CAC | 208 | 118 | 33.05 | 31 |
|  | 6 | m | ACT | CAG | 219 | 129 | 27.13 | 31 |
|  | 6 | m | ACT | CCA | 292 | 131 | 35.11 | 31 |
|  | 6 | m | ACT | CCT | 210 | 114 | 28.95 | 31 |
|  | 6 | m | ACT | CTC | 217 | 120 | 26.67 | 32 |
|  | 6 | m | ACT | CTG | 246 | 137 | 32.85 | 32 |
|  | 6 | m | AGG | CAA | 179 | 95 | 20 | 31 |
|  | 6 | m | AGG | CAT | 135 | 93 | 25.81 | 7 |
|  | 6 | m | AGG | CTA | 102 | 61 | 22.95 | 15 |
|  | 6 | m | AGT | CTC | 126 | 91 | 19.78 | 8 |
|  | 6 | m | ATC | CAG | 268 | 137 | 39.42 | 26 |
|  | 6 | m | ATG | CAC | 137 | 95 | 31.58 | 5 |
|  | 6 | m | ATG | CAG | 520 | 127 | 40.16 | 284 |
|  | 6 | m | ATG | CGA | 177 | 119 | 26.89 | 12 |
|  | 6 | m | ATG | CGT | 116 | 81 | 25.93 | 7 |
|  | 6 | m | ATG | CTC | 150 | 91 | 35.16 | 8 |
|  | 6 | m | ATG | CTG | 179 | 126 | 25.4 | 7 |
|  | 6 | GC | ACG | CAC | 76 | 64 | 12.5 | 4 |
|  | 6 | GC | ACG | CGC | 63 | 46 | 21.74 | 9 |
|  | 6 | GC | ACG | CTC | 230 | 43 | 6.98 | 180 |
|  | 6 | GC | ACT | CCC | 259 | 97 | 31.96 | 78 |
|  | 6 | GC | AGC | CGC | 53 | 42 | 14.29 | 9 |
|  | 6 | GC | AGC | CGT | 84 | 67 | 10.45 | 8 |
|  | 6 | GC | AGC | CTG | 106 | 82 | 18.29 | 6 |
|  | 6 | GC | AGG | CAC | 59 | 51 | 15.69 | 2 |
|  | 6 | GC | AGG | CAG | 58 | 48 | 12.5 | 5 |
|  | 6 | GC | AGG | CGC | 44 | 33 | 21.21 | 9 |
|  | 6 | GC | AGG | CGT | 51 | 37 | 16.22 | 9 |
|  | 6 | GC | AGG | CTC | 53 | 45 | 8.89 | 5 |
|  | 6 | GC | AGG | CTG | 85 | 72 | 11.11 | 4 |
|  | 6 | GC | ATG | CGC | 105 | 81 | 19.75 | 7 |
|  | 7 | AT | AAC | CGAT | 59 | 44 | 15.91 | 6 |
|  | 7 | AT | AGC | CAAT | 57 | 48 | 8.33 | 6 |
|  | 7 | AT | AGC | CATA | 46 | 40 | 10 | 4 |
|  | 7 | AT | AGT | CTAA | 86 | 62 | 22.58 | 7 |
|  | 7 | AT | AGT | CTCT | 33 | 31 | 6.45 | 2 |
|  | 7 | AT | ATA | CCAT | 70 | 52 | 17.31 | 7 |
|  | 7 | AT | ATA | CTAC | 63 | 47 | 17.02 | 5 |
|  | 7 | AT | ATA | CTGG | 33 | 27 | 11.11 | 5 |
|  | 7 | GC | AAC | CAGC | 57 | 39 | 17.95 | 6 |
|  | 7 | GC | ACA | CCAG | 17 | 17 | 0 | 1 |
|  | 7 | GC | ACC | CAGT | 32 | 24 | 8.33 | 6 |
|  | 7 | GC | AGC | CACG | 21 | 17 | 17.65 | 3 |
|  | 7 | GC | AGT | CTGG | 35 | 28 | 14.29 | 3 |
|  | 8 | AT | ACAA | CTAC | 4 | 3 | 33.33 | 2 |
|  | 8 | AT | ACAA | CTGA | 31 | 28 | 10.71 | 2 |
|  | 8 | AT | ACAA | CTTA | 6 | 6 | 0 | 1 |
|  | 8 | AT | ACAT | CCAT | 8 | 8 | 0 | 1 |
|  | 8 | AT | ACAT | CGAT | 11 | 11 | 0 | 1 |
|  | 8 | AT | AGAT | CGTA | 7 | 7 | 0 | 1 |
|  | 8 | AT | AGAT | CTAC | 9 | 8 | 12.5 | 1 |
|  | 8 | AT | AGAT | CTTA | 5 | 5 | 0 | 1 |
|  | 8 | AT | AGCT | CTAT | 6 | 6 | 0 | 1 |
|  | 8 | AT | ATCT | CATG | 17 | 15 | 13.33 | 2 |
|  | 8 | m | ACAA | CGCT | 6 | 6 | 0 | 1 |
|  | 8 | m | AGAT | CGCT | 10 | 10 | 0 | 1 |
|  | 8 | m | AGTC | CCAT | 5 | 5 | 0 | 1 |
|  | 8 | m | AGTC | CGAT | 14 | 12 | 8.33 | 3 |
|  | 8 | m | ATCT | CTGG | 9 | 9 | 0 | 1 |
|  | 8 | GC | AGTC | CCTG | 4 | 3 | 33.33 | 2 |
|  | 8 | GC | AGTC | CGGA | 6 | 6 | 0 | 1 |
|  | 8 | GC | ATCC | CCCT | 11 | 11 | 0 | 1 |
|  | 8 | GC | ATCG | CCAG | 3 | 3 | 0 | 1 |
|  | 8 | GC | ATCG | CGTC | 5 | 5 | 0 | 1 |

*^a^* Total number of selective bases added for the 2 primers.

*^b^* “GC cont” is composed of three classes of selective bases differing according to the proportion of their GC content: “AT” corresponds to selective bases containing a larger number of A or T, “GC” to selective bases containing a larger number of C or G, “m” to selective bases containing same number of A or T and C or G.

*^c^* Maximum number of co-migrating fragments in one single peak.
