# Supplementary material for: Amplified fragment length homoplasy: in silico analysis for model and non-model species
Source: BMC Genomics. 2010 May 7;11:287. doi: 10.1186/1471-2164-11-287 (PMC2875239; doi:10.1186/1471-2164-11-287)
Supplement: Additional file 2 — Table S2. Primer combinations and sample sizes used for each model/non-model species comparison and number of in silico and empirical peaks obtained. [file 1471-2164-11-287-S2.DOC]

Additional file 2 - Table S2.-Primer combinations and sample sizes used for each model/non-model species comparison and number of *in silico* and empirical peaks obtained.

| **Pairs of model - non-model species** | **EcoRI selective bases** | **PstI selective bases** | **MseI selective bases** | **No. of *in silico* peaks on model species** | **No. of empirical peaks on non-model species** | **No. of non-model species samples** |
| --- | --- | --- | --- | --- | --- | --- |
| *Bacillus thuringiensis* ser. *konkukian - Bacillus thuringiensis ser. israelensis* | - |  | A | 259 | 180 | 2 |
|  | - |  | C | 205 | 167 | 2 |
|  | - |  | G | 160 | 119 | 2 |
|  | A |  | A | 102 | 111 | 2 |
|  | A |  | C | 85 | 85 | 22 |
|  | A |  | G | 47 | 35 | 23 |
| *Arabidopsis thaliana - Arabis alpina* | AAT |  | CAC | 21 | 25 | 728 |
|  | AGC |  | CAC | 7 | 24 | 728 |
|  | ATC |  | CAC | 9 | 27 | 728 |
|  | AGG |  | CAC | 8 | 21 | 728 |
|  | ACG |  | CAG | 7 | 23 | 728 |
|  | AGC |  | CTG | 8 | 25 | 728 |
|  | ACG |  | CTC | 6 | 27 | 728 |
|  |  | AAG | CA | 66 | 62 | 728 |
|  |  | ACT | CA | 32 | 50 | 544 |
|  |  | ATC | CA | 21 | 44 | 543 |
|  |  | AAC | CT | 23 | 44 | 123 |
|  |  | AGA | CG | 23 | 36 | 123 |
|  |  | ACA | CA | 35 | 50 | 123 |
|  |  | AAC | CA | 47 | 52 | 728 |
|  |  | AGA | CA | 77 | 65 | 728 |
|  |  | ACA | CG | 12 | 52 | 728 |
|  |  | AAC | CG | 17 | 30 | 335 |
|  |  | AGA | CT | 61 | 65 | 329 |
|  |  | ACA | CT | 28 | 55 | 324 |
| *Aedes aegypti - Aedes rusticus* | AAC |  | CAA | 172 | 144 | 2 |
|  | AAC |  | CAC | 95 | 142 | 2 |
|  | AAC |  | CTC | 114 | 123 | 2 |
|  | AAC |  | CTG | 148 | 142 | 2 |
|  | ACA |  | CACTAG | 9 | 32 | 3 |
|  | ACA |  | CAT | 121 | 114 | 279 |
|  | ACG |  | CAC | 64 | 68 | 168 |
|  | ACG |  | CGC | 46 | 49 | 3 |
|  | ACT |  | CAA | 203 | 127 | 3 |
|  | ACT |  | CAC | 118 | 146 | 2 |
|  | ACT |  | CTC | 120 | 126 | 2 |
|  | ACT |  | CTG | 137 | 118 | 3 |
|  | AGC |  | CGC | 44 | 55 | 3 |
|  | AGC |  | CTG | 82 | 75 | 3 |
|  | AGT |  | CTC | 91 | 55 | 3 |
|  | ATA |  | CAA | 187 | 169 | 2 |
|  | ATA |  | CAC | 105 | 117 | 279 |
|  | ATA |  | CACTAG | 18 | 26 | 2 |
|  | ATA |  | CTC | 94 | 123 | 3 |
|  | ATA |  | CTG | 149 | 118 | 3 |
